# Excessive Neutrophil Activity in Gestational Diabetes Mellitus: Could It Contribute to the Development of Preeclampsia?

**DOI:** 10.3389/fendo.2018.00542

**Published:** 2018-09-21

**Authors:** Lenka Vokalova, Shane V. van Breda, Xi Lun Ye, Evelyn A. Huhn, Nandor G. Than, Paul Hasler, Olav Lapaire, Irene Hoesli, Simona W. Rossi, Sinuhe Hahn

**Affiliations:** ^1^Department of Biomedicine, University and University Hospital Basel, Basel, Switzerland; ^2^Institute of Physiology, Faculty of Medicine, Comenius University, Bratislava, Slovakia; ^3^Department of Rheumatology, Kantonsspital Aarau, Aarau, Switzerland; ^4^Department of Obstetrics, University Women's Hospital Basel, Basel, Switzerland; ^5^Systems Biology of Reproduction Momentum Research Group, Research Centre for Natural Sciences, Institute of Enzymology, Hungarian Academy of Sciences, Budapest, Hungary

**Keywords:** pregnancy, neutrophils extracellular traps, gestational diabetes, preeclampsia, TNFalpha, leptin, A1AT, elastase

## Abstract

Gestational diabetes mellitus is a transient form of glucose intolerance occurring during pregnancy. Pregnancies affected by gestational diabetes mellitus are at risk for the development of preeclampsia, a severe life threatening condition, associated with significant feto-maternal morbidity and mortality. It is a risk factor for long-term health in women and their offspring. Pregnancy has been shown to be associated with a subliminal degree of neutrophil activation and tightly regulated generation of neutrophil extracellular traps (NETs). This response is excessive in cases with preeclampsia, leading to the presence of large numbers of NETs in affected placentae. We have recently observed that circulatory neutrophils in cases with gestational diabetes mellitus similarly exhibit an excessive pro-NETotic phenotype, and pronounced placental presence, as detected by expression of neutrophil elastase. Furthermore, exogenous neutrophil elastase liberated by degranulating neutrophils was demonstrated to alter trophoblast physiology and glucose metabolism by interfering with key signal transduction components. In this review we examine whether additional evidence exists suggesting that altered neutrophil activity in gestational diabetes mellitus may contribute to the development of preeclampsia.

## Introduction

Gestational diabetes mellitus (GDM) is defined as glucose intolerance manifesting during pregnancy in women with no prior history of diabetes (1–4). It is, thus, by definition unlike either Type 1 or Type 2 diabetes mellitus (T1DM and T2DM, respectively), in that it is of a transient nature and gender specific. Furthermore, it only occurs during a unique physiological condition, namely pregnancy (1–4).

GDM does, however, share a number of traits with T2DM. These include insulin resistance or aspects of the metabolic syndrome (1–4). Due to its transient appearance in pregnancy, GDM has been suggested to be a pre-diabetic state or a momentary unmasking of a T2DM-like condition triggered by gestation (1–4). Unlike T1DM, there is no auto-immune component in GDM.

Although a number of strategies exist to manage pregnancies affected by GDM, these are nevertheless associated with several fetal and maternal complications (2, 5). These comprise fetal macrosomia, necessitating cesarean delivery, as well as an increased risk for severe complications such as preeclampsia (PE) (6). Post-partum complications can include neonatal hypoglycaemia, jaundice or respiratory distress syndrome. The condition triggers as to yet to be defined epigenetic alterations in mother and child, resulting in an increased risk for development of T2DM in both in the post-partum period (7, 8).

Current screening protocols rely on a glucose challenge test (GCT), also termed oral glucose tolerance test (OGTT), generally performed in the 2nd trimester of pregnancy (9). This test can also be used to stratify the degree of severity of GDM, and thereby provide a guide for the route of therapy to be taken. In severe cases this may include treatment with insulin or metformin, whilst less severe forms are usually managed by a regimen of diet and exercise (2, 3, 10, 11).

The underlying etiology of GDM has yet to be determined, but previous studies suggest that it may involve a contra insulin effect mediated by placentally produced hormones and cytokines (12, 13). It is further proposed that this condition may be exacerbated by high calorie diet, obesity, or genetic predisposition prevalent in certain ethnicities (3).

Since obesity and the associated metabolic syndrome have a dramatic effect on pregnancy (14), the significant rise in the prevalence of this condition can be expected to lead to an increase in the number of pregnancies affected by GDM. Consequently this will lead to an increase in associated complications, such as PE (15, 16). This demographic change could have a seismic shift-like impact on health care systems, both in terms of a cost explosion and additionally straining already short stretched staff resources (16).

## PE and diabetes—a complex relationship

PE is a severe disorder of pregnancy, characterized by hypertension, proteinuria, oedema and multiple organ distress, in previously normotensive women (17–19). PE typically affects between 3 and 8% of all pregnancies, having a higher prevalence in certain ethnicities (6).

Despite a relatively high prevalence in pregnancy, PE has recently been classified as an “orphan disease” on account of its low incidence in the entire population; a feature which will hopefully enable the accruement of additional research funding (20).

PE typically arises in the 2nd or 3rd trimester of pregnancy, but can also occur post-partum (21). If left untreated, PE advances to eclampsia, a condition characterized by epilepsy-like seizures and associated with high rates of maternal mortality (22). PE is also a significant risk factor for subsequent pregnancies, as well as the long-term health in women and their offspring.

The underlying etiology of PE is complex and multifactorial, more akin to a syndrome than a singular disease (17–19, 23). Evidence suggests that the lesion initiating the cascade of events leading to the manifestation of clinical symptoms occurs early in pregnancy (17–19, 24). PE therefore seems to involve a long asymptomatic phase, the appearance of symptoms depending on the accrual of secondary or tertiary hits (17–19).

On account of its complex underlying etiology, it has been suggested that PE can be stratified into an early onset (eoPE; <34 weeks) and late onset form (loPE; ≥ 34 weeks), according to the time of onset during gestation, and that these two forms can be viewed as disparate entities, akin to the segregation of diabetes mellitus into Type 1 and T2 forms (25).

EoPE is associated with more severe symptoms and poorer feto-maternal outcome, particularly due to premature delivery of fetuses, many of which are growth restricted (25). In general, the early form of PE is less prevalent than the late form (loPE), by a proportion of approximately 1:10 (6, 22). Both forms of PE have made the quest to develop suitable biomarkers to detect at risk pregnancies a challenging task (26).

Key features of PE include abnormal placentation, particularly aberrant modification of the maternal spiral arteries, altered expression of key angiogenic and anti-angiogenic factors, elevated feto-maternal cell trafficking, release of placentally-derived cell-free DNA and occurrence of neutrophil extracellular traps (NETS) in the intervillous space of affected placentae (18, 26–29). These features vary between early and late onset forms, with placental deficiencies, such an inadequate modification of the spiral arteries and infarction, being more pronounced in cases with eoPE than in the late onset form (18, 26). On the other hand, maternal inflammation appears to be a key contributor to the development of loPE, because it can be triggered by extraneous influences such as air pollution or obesity (15, 26, 30).

Although diabetes in pregnancy is frequently associated with poor outcome, it poses a significant increased risk for the development of PE (6, 31, 32). This is particularly high in instances with pre-existing T1DM, where 20% or more of such pregnancies can develop PE (31). The presence of T2DM prior to conception is also associated with a significant increase (2–4 fold) in the development of PE (31).

Although less dramatically than in cases with T1DM, pregnancies affected by GDM incur a higher risk of developing PE. The aetiological trigger leading to the onset of PE by either pre-existing diabetes or GDM is currently unclear (31).

To date few studies have addressed the association of diabetic conditions with the development of either early or late-onset forms of PE. Probably the most salient study was performed by Lisonkova and Joseph, who analyzed risk factors associated with the development of either eoPE or loPE in a very large cohort (*n* = 456668) (6).

In this study, the overall incidence of PE was 3.1%, of which 0.38% was affected by eoPE and 2.72% by loPE (6). Risk factors for eoPE were determined to include ethnicity (African-American), chronic hypertension and congenital anomalies. On the other hand, young maternal age, nulliparity and diabetes prior to conception were determined to pose a significant risk for the manifestation of loPE. In this study the issue of GDM and the incidence of PE was unfortunately not addressed (6).

The role of GDM in the development of PE was recently examined in 120 pregnant women, of whom 60 each had early-onset and late-onset GDM (33). The results indicated that early onset GDM was associated with a significantly higher incidence of PE (23.3%) compared with 10% in the late-onset group. Early onset GDM was also determined to be more severe, requiring a greater amount of therapeutic intervention with agents such as insulin. Unfortunately, no clear indication was given regarding the stratification of eoPE and loPE (33).

The interplay between PE, diabetes, or GDM is however not restricted to the current pregnancy, but may influence the course of subsequent pregnancies. In this manner, recent data indicated that PE in a previous pregnancy increases the risk of GDM in a subsequent pregnancy (34). This was even more pronounced in cases when the previous pregnancy was affected by both GDM and PE (34).

PE and GDM may also contribute to other pathologies post-partum. It has been demonstrated that PE leads to an increased risk of developing T2DM in previously non-diabetic women; a feature also evident post-partum in women affected by GDM (35, 36). The incidence of post-partum diabetes, however, appears to be significantly higher in cases affected by GDM (19%) than those by PE (2%), as suggested by a large-scale epidemiological study (36).

In cases with a pre-existing diabetic condition, the effects of ensuing PE can be far reaching by contributing to the post-partum development of retinopathy or nephropathy (31). It is also well established that both GDM and PE are associated with an increased risk for the post-partum development of cardiovascular pathologies (22, 37, 38).

## GDM and PE—the contribution of obesity

A causal relationship between obesity and insulin resistance in T2DM is historically well established, and includes pioneering observations that insulin levels were greater in obese diabetic individuals than lean healthy counterparts (39, 40). A key contribution into understanding this interaction was by uncovering a low-grade chronic inflammation mediated by metabolic cells in response to nutrient overload (39, 40). Hence, the so-called metabolic syndrome provided an important link between obesity, sedentary lifestyle, stress, and onset of T2DM.

The initial observation of an inflammatory event triggered by diet was made in adipose cells, where obesity was determined to trigger TNFα production (41, 42). This observation was soon extended to show that a host of tissues were affected by nutrient overload including liver, pancreas, brain and muscle, involving the pro-inflammatory cytokines including IL-6, IL-1β, and CCL2 (39, 40). The inflammatory cascade appears to involve the infiltration of affected tissues, such as adipose tissue, by macrophages and other regulatory immune cells in obesity. The underlying molecular process is suggested to involve the activation of Toll-like receptors (TLRs) and the inflammasome by circulating free fatty acids (FFAs), resulting in the production of IL-1β or TNFα, which promote tissue infiltration and immune cell activation. Furthermore, in the context of T2DM, these highly pro-inflammatory cytokines have the ability to dampen or hinder insulin signaling, hence their original description as *insulin antagonists* (39, 40).

As mentioned above, obesity has been suggested to fuel the increase in both GDM and PE (3, 15). It is noteworthy that GDM is not restricted to women with an elevated BMI, while not all overtly obese women develop PE. This is most evident when comparing the incidence of GDM in various population groups, for instance in the Indian women from the Indian sub-continent who have an 11 fold greater risk for glucose intolerance during pregnancy than their Caucasian counterparts (43). The aspect of race or ethnicity was more extensively examined by Hedderson et al. in a cohort of 216 089 pregnant women (44). Their results indicated that the incidence of GDM was lowest in Caucasian women (4.1%) and highest in Asian Indians (11.1%) (43). Obesity was determined not to correlate significantly with the incidence of GDM in Asian Indian migrant populations (45, 46).

On the other hand, obesity has profound effects during pregnancy, and has been shown to affect fetal development, enhancing the risk for macrosomia, fetal defects and preterm labor, independent of the occurrence of either GDM or PE (3, 16).

That obesity can directly contribute to the onset of PE was strikingly shown by Mbah et al., in a study where they examined the outcome of over one million live births (15). They noted that an increase in BMI was associated with an almost exponential increase in the incidence of PE. This was most striking when examining cases with super obesity with a BMI > 50, where an almost 4 fold increase in PE was noted (15).

A key feature of this analysis was the stratification of PE into early and late onset forms, which clearly indicated that increased BMI lead to a significant increase in late-onset PE, whereas only a modest increase in eoPE was noted (15).

This would appear to support the tenet that loPE is promoted by maternal inflammation, while eoPE appears to result from underlying placental dysfunction (17, 26). It would thus seem that an underlying inflammation in loPE triggered by obesity, such as is evident in the metabolic syndrome, may be a causative feature promoting the occurrence of loPE associated with elevated BMI (26).

These datasets suggest that the overall scenario is quite complex, and that underlying genetic propensities in combination with environmental factors, interact in rendering pregnant women susceptible to the advent of GDM, PE or both, by obesity (17, 26).

## GDM and PE: underlying placental changes

Numerous reports indicate that the placenta is adversely affected by GDM (12, 47). Poorly controlled diabetes can result in gross morphological aberrancies such as an enlarged, thickened plethoric placenta, with reduced fetal-placental weight ratio. In addition, calcification and other features associated with GDM can be detected by sophisticated ultrasound examinations (12, 47).

GDM can also influence villous development, resulting in villous immaturity, aberrant villous branching and hyper-vascularization of the villous tissue. In addition, turnover of the villous tissue is altered resulting in an abnormally thin syncytiotrophoblast layer, which is nearly devoid of nuclei. This may be due to reduced apoptosis in the underlying villous trophoblast tissue (12, 47).

These alterations could have a pronounced effect of the release of placental micro-debris by the trophoblast. Commonly these syncytiotrophoblast—derived microparticles are referred to as STBM (48). As we will see below, STBM have long been implicated in the etiology of PE, and may hence provide for a link between GDM and the enhanced occurrence of PE in affected pregnancies. Additionally, increased expression of IL-1β and TNFα may occur in GDM placentae, which could profoundly influence the behavior of maternal immune effector/regulator cells in this milieu (49, 50). These cytokines could also promote insulin resistance by antagonizing insulin signaling (41).

A generally accepted canonical view is that the placenta plays a key role in the development of PE, since most cases of PE are resolved following delivery and removal of the placenta (18). Furthermore, PE can occur in hydatidiform molar pregnancies that consist exclusively of trophoblast tissue. An important placental anomaly associated with PE, especially eoPE, is the inadequate modification of maternal spiral arteries by invasive cytotrophoblast cells (18, 51).

This defect appears to be a consequence of placental development during human pregnancy (52), which is unique in having a very deep form of placentation. Additionally, the placenta is subject to significant changes in oxygen tension during the course of gestation. This is especially evident during early embryo development, where there is limited contact of the placenta with the maternal circulation, leaving most of the fetal tissues in a hypoxic state (52). The reason for limited feto-maternal contact is due to the blockage of maternal spiral arteries by fetal cytotrophoblast cells. This blockage is suggested to protect the fetus from the teratogenic action of toxic reactive oxygen species during this crucial stage of development. By the end of the first trimester these plugs are gradually removed and subsequently the spiral arteries are widened by the action of invasive cytotrophoblast cells, permitting an even high capacity flow of maternal blood to the underlying fetal tissues (52).

Failure of this modification is frequently high-lighted as being a key placental anomaly occurring in PE (18, 51). Such an anomaly is however not restricted to PE and is frequently evident in cases affected by IUGR (intra-uterine growth restriction) and to a lesser extent in cases with idiopathic preterm delivery or premature preterm rupture of membranes (51). Further, it is evident that these placental defects are less common in cases with loPE than eoPE (18).

PE is sometimes referred to as pregnancy toxemia, in the context of which pioneering studies by the Oxford group of Redmond and Sargent, identified a candidate for such a noxin in the form of placental micro-debris, specifically the STBM (syncytiotrophoblast microparticles) alluded to above (48). These STBM are released by the turn-over of the syncytiotrophoblast, the large multinucleate single cell layer covering the entire villous tree. During their studies they noted that STBM release into the maternal circulation was elevated in cases with manifest preeclampsia (48). Subsequent studies indicated that STBM were highly pro-inflammatory, capable of activating maternal immune cells or having a deleterious effect on maternal endothelium (48, 53).

## GDM and PE: the issue of placental mass

It is currently not clear how pregnancy triggers the onset or development of GDM. It does, however, appear that multiple fetuses, and therefore placental mass, could be a key component. This is evident from studies on multi-fetal pregnancies, where a significant increase in glucose intolerance and GDM was noted compared to singleton pregnancies. The degree of GCT discordance was greatest in pregnancies with triplet fetuses, whilst it was still significantly altered in those with twins (54). An independent examination of pregnancies with triplet fetuses indicated that 10% were affected by GDM (55).

In a similar manner, PE is elevated in multi-fetal pregnancies and may be influenced by placental chorionicity (6). A recent report examining birth outcome in approximately 100,000 pregnancies, indicated that the incidence of PE in singleton pregnancies was 2.3%, 6% in monochorionic twin and 8.1% in dichorionic twin pregnancies (56). In triplet pregnancies the rate of PE can approach 20% (55). While not clear, there is some evidence that the incidence of PE may be higher in multi-fetal pregnancies conceived via assisted reproductive technologies (ART) than spontaneously (32). This could be related to major histocompatibility complex (MHC) differences between mother and fetus and lack of previous exposure to paternal antigens, which have been implicated in the development of PE (57, 58).

## Pregnancy is associated with a maternal inflammatory response: neutrophils enter the centre court

Neutrophils, also termed polymorphonuclear neutrophil granulocytes, are the most prevalent leucocytes in human circulation (59). They are an essential component of the primary immune response to microbial infection largely by phagocytic activity or by the release of cytotoxic granular enzymes such as myeloperoxidase (MPO) or neutrophil elastase (NE). The action of these is frequently enhanced by the concomitant release of reactive oxygen species (ROS) (59).

During their investigations into the action of STBM in normal pregnancy and those affected by PE, the Oxford group made the notable observation that normal pregnancy was associated with a subliminal inflammatory response (60). This was especially evident when examining maternal innate immune cells such as neutrophils. A pertinent and often overlooked aspect of these studies is that the activation status of neutrophils, as assessed by expression of CD11b and ROS production, was significantly greater in cases with PE than those with sepsis used as an inflammatory disease control (60). Therefore, PE was clearly associated with a highly inflammatory state, possibly initiated by the release of placental micro-debris.

Although there is still a paucity of data on the role of neutrophils in reproduction, they have been implicated in spiral artery remodeling via the action of Placental Protein 13 (PP13), as well as in recurrent fetal loss associated with anti-phospholipid antibodies [recently reviewed in (61)]. There is, however, no doubt that the key finding leading to a renewed interest in neutrophil biology is their ability to form neutrophil extracellular traps (NETs).

## Neutrophils in PE: leaving the baseline to storm the NET

As mentioned, a noteworthy aspect of neutrophil physiology is their ability to form extracellular traps (NETs), a process whereby they extrude their nuclear chromatin into the surrounding environment, via a process termed NETosis (62, 63). This process relies on a series of discrete events that include calcium mobilization, ROS production, nuclear translocation of granular enzymes (MPO and NE), and histone citrullination by peptidylarginine deaminase 4 (PAD4) (64, 65). NETs were originally described as a novel tool to ensnare and kill invasive pathogens, a process facilitated by the adhesion of bacteriocidal granular proteins to the DNA lattice structures (63). They have, however, in the interim become implicated in the underlying etiology of a number of human pathologies, including PE (29, 66).

Our interest in NETs stems from our studies into the use of cell-free DNA in maternal blood for non-invasive prenatal diagnosis of fetal aneuploidies and Mendelian disorders (67). During the course of these analyses we had observed that the levels of cell-free DNA were significantly elevated in cases with manifest PE (68). A unique aspect of our analyses was that we examined for the quantity of both placentally-derived fetal cell-free DNA, as well as that of maternal origin (68). This examination indicated that there was a reciprocal elevation in the quantity of both cell-free DNA entities, which corresponded to severity of PE symptoms (68).

By examining samples collected early in pregnancy prior to onset of PE symptoms, we furthermore observed that in asymptomatic conditions, only the levels of placentally-derived fetal cell-free DNA were elevated in those pregnancies which subsequently developed PE (69). This provided yet further evidence of an initiating placental lesion occurring early in gestation, weeks prior to manifestation of maternal symptoms (70).

As the origin of maternal cell-free DNA was unclear, particularly the elevated quantities in PE, we were intrigued by the discovery of NETs and questioned whether these new entities could be a potential source (71). It should be noted there was no indication that NETs could be involved in human pathology at the time.

Prompted by the findings of the Oxford group, we examined the activity of STBM on isolated neutrophils, wherein we confirmed that these were activated by STBM, as assessed by increased expression of CD11b and generation of ROS (29). To our amazement, we clearly observed the generation of NETs following STBM treatment. We were furthermore able to readily detect the abundant presence of NETs directly in the intervillous space of affected placentae (29); thereby underscoring the possible involvement of such a process in the underlying etiology of PE. Based on the overt presence of these lattice structures in the intervillous space we hypothesized that they could facilitate placental hypoxia or ischemia, features associated with PE (52, 72). This presented the first report indicating that NETs could contribute to a human pathology, a feature that has since been reported in a wide host of disorders including rheumatoid arthritis, systemic lupus erythematosus, small vessel vasculitis, coagulopathies, diabetes, and possibly tumor growth (66).

We were subsequently able to show that NETs could induce apoptosis of adjacent cells, such as endothelial cells, indicating that they lead to considerable damage of surrounding placenta tissue (73). Using specific immuno-assays for NETs-derived products, we subsequently demonstrated that elevated maternal cell-free DNA molecules in the blood of women affected by PE were indeed of NETotic origin, thereby providing the necessary proof for our original hypothesis (71, 74).

Furthermore, in a translational study conducted together with the groups of Wagner and Karumanchi (Harvard, Boston, USA) it was determined that overt NETs formation could indeed contribute to PE-like symptoms or fetal loss in a murine model system (75). NETS clearly contributed to the development of these conditions, as they were absent in genetically modified mice incapable of undergoing NETosis. These data provide crucial additional credence to the hypothesis that overt neutrophil activity, particularly in the form of NETosis, can contribute to the development of severe complications of pregnancy, or even result in fetal loss (76).

## Complex multi-modal regulation of NETosis during the course of normal pregnancy

Since a mild inflammatory state occurs in human pregnancy characterized by neutrophil activation (60), we recently examined the NETotic response during the course of normal gestation (77). Our data indicated that during pregnancy circulatory neutrophils exhibited an enhanced propensity to undergo NETosis, which increased progressively during the course of gestation. This feature was mediated in part by the action of G-CSF (granulocyte colony stimulating factor), the levels of which increased concomitantly during pregnancy (77).

A fascinating observation made in this study concerned the multi-modulation of neutrophil activity and NETosis by pregnancy associated sex hormones. In this context, NETosis is enhanced by chorionic gonadotropin during the first trimester of pregnancy, whereas toward term a complex interaction arises, in that it is stimulated by estrogen, but restrained by progesterone. In these neutrophils, extensive histone citrullination is evident, yet despite the presence of this key requirement in the NETotic cascade, they are unable to progress to full NETs formation. This hindrance appears to be mediated via the inability of NE to migrate to the nucleus by the action of progesterone. Previous studies have indicated that nuclear localisation of NE is essential for chromatin decondensation via the proteolytic action of this enzyme on histones, particularly the linker activity of histone H1 (64).

In this manner, progesterone appears to have a unique ability to regulate a vital step required for NETosis, maintaining circulatory neutrophils in a highly primed pro-NETotic, yet restrained state, ready to respond immediately and vigorously to an infection. Furthermore, as both phagocytosis and degranulation by neutrophils are enhanced in pregnancy, our data indicate that the innate arm of the immune system is highly active during the course of gestation to safeguard maternal and fetal wellbeing.

A possible downside of maintaining such a primed pro-active state is that it may be highly susceptible to inappropriate NETotic triggering by aberrant conditions. This could lead to the initiation of PE. Such an event could occur in pregnant women with systemic lupus erythematosus, where flares are strongly associated with the development of PE (78, 79). This supposition is supported by a recent report describing the presence of NETs in placentae of such cases with lupus induced PE (80).

## NETs in diabetes: bystanders or contributors to associated pathologies?

Recent reports indicated that neutrophil activity, specifically NETosis, may be altered in diabetes [reviewed in (81)]. Unfortunately there was some confusion in initial reports as to whether NETosis was enhanced or reduced under diabetic or hyperglycemic conditions (81). Despite this preliminary confusion, these reports, however, may provide insight into the associated pathologies, such as reduced wound healing ability or increased susceptibility to bacterial infections in diabetes.

In an initial study examining NETosis in diabetes, it was suggested that the increased prevalence of infections with *Burkholderia pseudomallei* and resulting in diabetic patients was due to a defect in NETosis (82). It was noted that neutrophils isolated from diabetic patients exhibited a reduced ability to generate NETs following stimulation with phorbol ester, usually a powerful trigger of the NETotic cascade. This translated into a diminished ability by neutrophils from diabetic cases to trap and kill *B. pseudomallei* micro-organisms (82).

A similar observation was made by Joshi et al., who determined that although spontaneous NETosis was enhanced under hyperglycaemic conditions, it was reduced when such neutrophils were stimulated with lipopolysaccharides (LPS) (83). In addition, in a recent report by Raposo-Garcia et al., it was observed that neutrophils and macrophages from individuals with T2DM displayed a reduced capacity to engulf and destroy *Mycobacterium tuberculosis* bacilli (84). These findings provide compelling evidence that neutrophil activity is altered in diabetes, and could serve to explain the increased sensitivity of these patients to infections.

In contrast, however, Menegazzo et al., using neutrophils isolated from healthy donors, determined that NET formation was enhanced by hyperglycaemia (25 mM glucose), and further increased following stimulation with phorbol ester (85). These authors furthermore detected evidence for increased NETosis in T2DM patients by the examination of surrogate markers for NETosis in plasma.

Altered neutrophil activity may also contribute to other pathologies associated with diabetes. In this context, Wong et al. argued that aberrant NETosis in diabetes (T2DM) could contribute to impaired wound healing (86). In their seminal study, they observed that diabetes promoted enhanced NETs formation, which was further increased by calcium influx promoted ionomycin (86). This facet was linked to increased expression of PAD4 in neutrophils from diabetic individuals. As discussed above, this enzyme is required for histone citrullination, a key step initiating chromatin decondensation, preparing the cell for subsequent NETosis (65). In a murine model system, they observed that tissue wounding lead to a pronounced influx of NETting neutrophils, a feature more pronounced under diabetic conditions (86). In their experimental setting wound healing was significantly increased in both normal and diabetic mice in which the PAD4 had been “knocked-out” by genetic ablation, indicating NETs involvement.

These findings were largely corroborated by an extensive investigation of diabetic foot ulcers by Fadini et al., who detected NETs-derived products in affected human tissue specimens (87). In a murine model ulcer formation and healing could be improved by pharmacological inhibition of PAD4 enzyme activity.

In summary, these data provide compelling evidence that aberrant NETosis in diabetes could contribute to associated pathologies such as increased susceptibility to infection and reduced wound healing capability. They also provide tempting therapeutic approaches, such as the use of PAD4 inhibitors to modulate NETosis in order to address these current issues.

## Not only NETs: exogenous neutrophil elastase usurps IRS1 signaling

In a hallmark study, Houghton et al. determined a possible mechanism whereby infiltrating neutrophils contribute to tumor growth (88). The basis for this study was the finding that an inflammatory milieu has a profound influence on tumor growth (89, 90). This appears to be largely due to infiltrating immune effector cells, including macrophages and neutrophils, facilitated by tumor chemokine release. These reports also indicate that auspicious neutrophil infiltrates are associated with poor prognosis (89, 90).

In a murine model system for lung cancer, NE significantly influenced tumor growth and murine survival (88). The most notable aspect of this analysis was that none of the mice died in which the NE gene (*Elane*) had been ablated, whilst all mice with an intact *Elane* gene had demised during the 30 weeks study period. Furthermore tumor size and burden was also significantly higher in mice with an intact *Elane* gene than ablated counterparts.

To gain insight into the underlying mechanism the authors examined the interaction between PMN and tumor cell lines. Here they made the key finding that NE released by infiltrating neutrophils was sequestered into neighboring tumor cells, enhancing proliferation. The authors determined that once internalized this potent proteolytic enzyme lead to the degradation of IRS1 (insulin receptor substrate 1), a key regulator of the mitogenic pathway triggered by PDGF. In a series of experiments they demonstrated that removal of IRS1 in the tumor cells line investigated lead to unrestricted growth factor independent mitosis, whereas the overexpression of IRS1 or inhibition of NE proteolytic activity reduced proliferation. Hence, infiltrating neutrophils appear to promote tumor growth by the uptake of exogenous NE, via the uncoupling of a key regulatory pathway.

These findings paved the way for similar analyses in other pathologies, including diabetes. In T2DM, evidence for such a mechanism was provided by Talukdar et al. (91), by observing that hepatocytes treated with exogenous NE became insulin resistant. They furthermore observed that mice fed a high fat diet (HFD) had improved insulin resistance and glucose tolerance, when the NE gene was ablated. Akin to the observation made in cancer cells, it was determined that exogenous uptake of NE by liver cells interfered with IRS1 signaling (91).

Subsequently, in an in-depth translational study, Mansuy-Aubert et al., observed increased activity of NE in obese human individuals. This feature was also evident in HFD obese mice, underscoring the value of this model system. Of note was that increased NE activity was coupled with a reciprocal reduction in the level of the NE inhibitor, alpha-1 antitrypsin (A1AT). This imbalance would increase enzymatic activity of liberated NE. In knockout and transgenic animals they obtained additional evidence that NE plays a key role in obesity induced insulin resistance in that NE knockout mice were resistant to HFD. This feature was also evident when NE activity was blocked pharmacologically, or by overexpression its natural inhibitor, A1AT, in transgenic mice.

## Neutrophils and GDM: interplay between hyperglycaemia, TNFα, and exogenous elastase

In our analysis of neutrophil activity during normal pregnancy discussed above (77), we noted an unprecedented degree of NETosis in a sample, that we assumed to be have been drawn from a normal healthy pregnant woman. A reappraisal of the case history indicated that it was from a pregnancy affected by GDM, a facet diagnosed after to the time of sample collection (92).

Intrigued, we set out to verify this phenomenon in a larger cohort, the analysis of which indicated that GDM was indeed associated with an excessive NETotic response when compared to matching healthy controls (92).

This was observed using both freshly isolated neutrophils, where we observed that GDM derived cells displayed a significantly increased propensity to undergo spontaneous NETosis; and by the use of surrogate serum markers (cell free nucleosomes and/or complexes with neutrophil granular proteins) for NET formation (74, 93), where increased levels of these analytes were detected in GDM cases (92).

We were moreover able to compare the NETotic response in cases with T1DM and T2DM to that in GDM, which indicated that this was greatest in the latter, intermediate in T1DM and lowest in T2DM (92). NETosis in all classes of diabetes was, however, significantly greater than in healthy matching controls; while only that in GDM exceeded basal levels in normal pregnancy. These data underscore potential differences between the various forms of diabetes based on neutrophil activity.

Our examinations indicated that high glucose conditions (25 mM) did indeed promote an enhanced level of NETosis in freshly isolated neutrophils, but that it on its own it could not account for the high levels evident in GDM.

In order to understand the mechanism leading to this very high level of NETosis in GDM, we made use of an *in-vitro* BeWo trophoblast cell culture system. Since previous studies suggested that the placenta produces TNFα in GDM (50), we examined the effect of hyperglycaemic (25 mM glucose) conditions on BeWo cells. Such treatment elevated TNFα production by BeWo cells. Additionally, appropriate culture supernatants exerted a pronounced pro-NETotic effect on freshly isolated normal neutrophils. Since we also determined that circulating levels of TNFα were also increased in the plasma of GDM cases, it appears that this potent pro-inflammatory cytokine renowned for its potential to prime neutrophils (94, 95), played a vital role in promoting the pro-NETotic phenotype occurring in GDM.

Since numerous other cytokines could be produced by the placenta under high glucose conditions, and by analogy BeWo cells, we sought to confirm that TNFα was responsible for the observed pro-NETotic effect. This was achieved using infliximab, a clinically employed biologic agent used to counter TNFα activity in auto-inflammatory diseases such as rheumatoid arthritis. The addition of infliximab was determined to reduce the pro-NETotic effect of both GDM sera and high-glucose BeWo culture supernatants. It therefore appears that TNFα plays a pivotal role in priming neutrophils for NETosis in GDM.

Interestingly, we detected a significantly increased neutrophil infiltration in GDM placentae, both by immunohistochemistry as well as quantitative PCR for NE mRNA. Granted recent findings concerning the interaction of exogenous NE with surrounding tissues we examined whether exogenous NE could influence trophoblast behavior akin to what had previously been observed in cancer cells or diabetic hepatocytes (88, 96). Our analysis of BeWo trophoblast cells indicated that exogenous NE lead to decreased expression of IRS1 (92). A decrease in the amount of this key regulatory protein was also observed in affected placentae, concomitant with an increase in elastase expression. Hence, our data appear to mirror the effect of exogenous on IRS1 expression observed in previous studies (92).

We furthermore determined that NE altered the glucose response of BeWo cells, by reducing the expression of GLUT4 glucose transporter protein. This led to a diminished ability of NE treated cells to respond to insulin, evident by a decrease in glucose uptake.

An important aspect of our study was that circulating levels of A1AT, the natural inhibitor of NE proteolytic activity, were decreased in GDM cases. Accordingly, the action of NE liberated by degranulation or NETosis could potentially be more vigorous than under normal conditions, where this activity is held in check by A1AT (92). As we shall see below, A1AT holds another trick or two up its sleeve.

## A1AT: elastase inhibitor or modulator of neutrophil activation?

A1AT, also termed serpin A1, is a protease inhibitor with a high specificity for neutrophil enzymes including NE, cathepsin G, and proteinase-3 (97, 98). It is probably best known for its deficiency (alpha-1 anti-trypsin deficiency/AATD) in affected individuals, where symptoms include chronic obstructive pulmonary disease (COPD), liver disease and in rare instances, panniculititis (97, 98).

AATD patients also exhibit other inflammatory characteristics, which cannot be derived merely from loss of protease inhibitor function. These include the ability to regulate the inflammatory action of IL-1β, IL-6, TNFα, and IL-8. On the other hand a number of studies using exogenous or transgenic A1AT have observed tolerogenic activities that cannot be attributed solely to protease inhibition (97, 98).

This was most strikingly observed when examining the action of a recombinant form of A1AT without elastase inhibiting properties in a murine pulmonary inflammation model, where it was as effective as normal functional A1AT (99).

**Figure 1:**
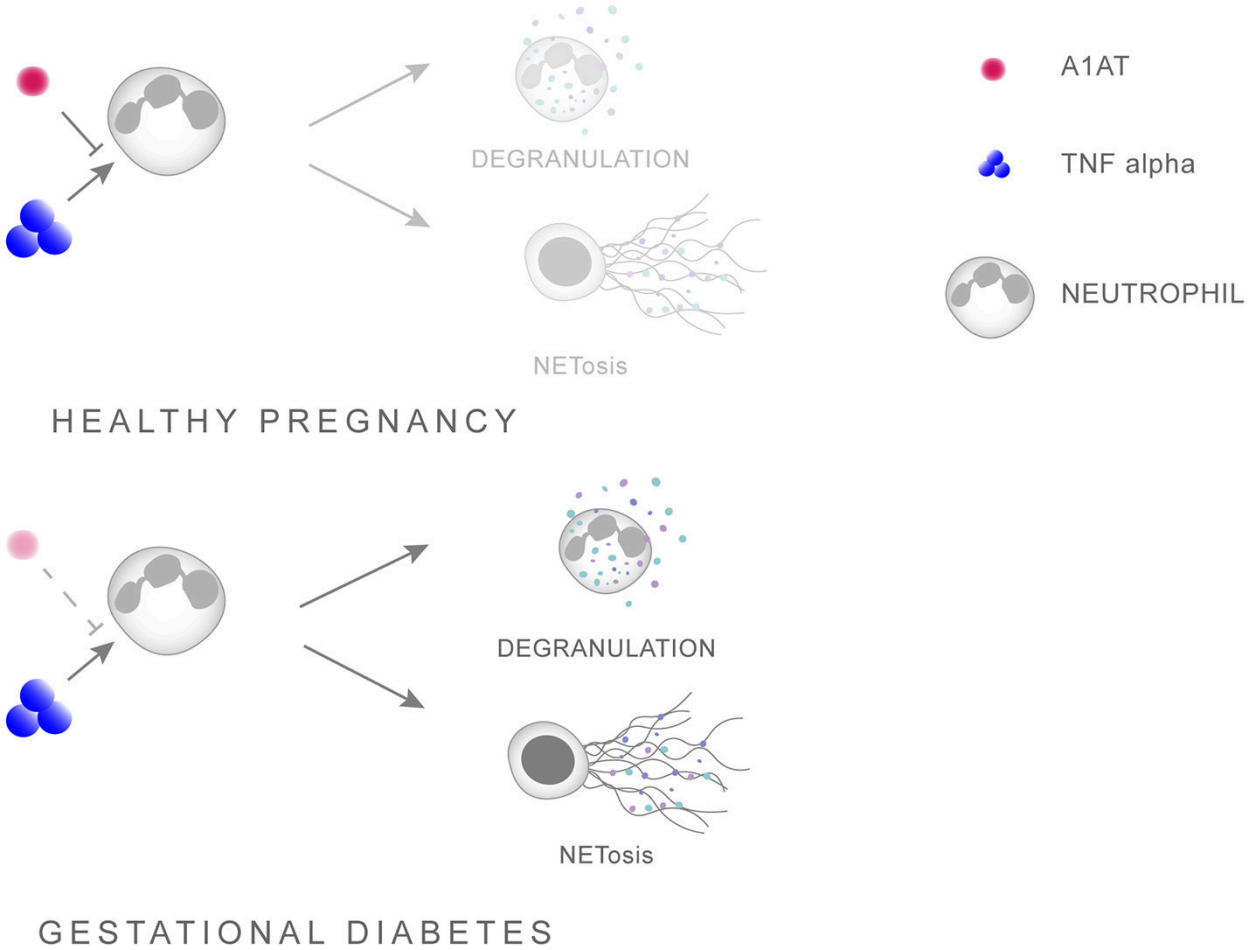
Scheme illustrating the interaction between A1AT and TNFα in regulating neutrophil priming. A1AT hinders the ability of TNFα to interact with its receptor, thereby reducing the extent of neutrophil priming and subsequent degranulation. In GDM this system is skewed in favor of overt neutrophil priming due to enhanced TNFα levels concomitant with reduced A1AT concentrations.

Further evidence of a pleiotropic activity by A1AT is that AATD is frequently associated with overt neutrophil activation in the absence of infection, being especially sensitive to the action of TNFα (97, 98). In this context, a recent report indicated that augmentation with A1AT in AATD individuals diminished neutrophil activity, by specifically reducing degranulation via affecting the binding of TNFα to its receptor [Figure 1; (100)].

Other reports indicating that A1AT possesses pleotropic activities, other than protease inhibition include the observation that exogenous A1AT reduces ROS production in stimulated neutrophils (101), or that A1AT disrupts neutrophil migration by binding to IL-8, a key chemokine (102). In addition, A1AT may blunt the immune response by binding to danger signals or damage associated molecular patterns (DAMPS) such as 70kDa heat shock protein (HSP70) (97).

As we have seen above, an imbalance between NE and A1AT has recently been implicated in obesity induced insulin signaling and energy metabolism, suggesting this pair maybe involved in the metabolic cascade initiating T2DM (96). This and other studies have prompted the exploration of recombinant A1AT molecules for the treatment of inflammatory cascades associated with the development of T2DM (103).

In the context of pregnancy, it is worth noting that circulatory A1AT levels increase during the course of gestation (97), while low circulating levels are associated with both recurrent and spontaneous abortion (104), as well as cases with severe PE (105). As the latter pathologies are associated with overt neutrophil activity (61, 76), it is currently unclear what the contribution of reduced A1AT levels is. An important facet concerning the regulatory action of A1AT in GDM, where we have noted a reduction in circulating A1AT levels, is that the activity of A1AT is diminished by high glucose conditions (97).

## Leptin—more than an adipokine?

Leptin, the satiety hormone, was originally described as a hormone produced by adipose tissue, regulating hunger and whose action is deregulated in obesity (106). In the interim leptin was established as an important molecule in immune regulation, most evident by virtue of its absence leading to an enhanced susceptibility to infection (106). Leptin has been described to trigger monocyte proliferation, activation, and release of pro-inflammatory cytokines, including TNFα (106).

Although neutrophils do express the short form of the leptin receptor (Ob-Ra), this is not sufficient to trigger their activation by leptin. Rather their activation by this cytokine is dependent on an interaction with monocytes and the presence of TNFα (106). Leptin has been shown to promote neutrophil migration, a feature attributed to the production of TNFα and CXCL1 by affected tissues (107). In addition leptin has been shown to enhance neutrophil longevity by suppressing apoptosis of mature neutrophils, which could be an important contributing factor for inflammatory processes (108). It is currently not clear if leptin modulates neutrophil functions, such as NETosis or degranulation.

Leptin plays a crucial role in pregnancy, where it is produced by placenta, in prodigious amounts (106, 109). In reproduction, leptin is implicated in the process of implantation, and during gestation it is implicated in regulating trophoblast differentiation, maturation and apoptosis, as well as the expression of key tolerogenic molecules such as HLA-G (106).

Leptin expression is enhanced in GDM placenta, apparently by the action of insulin or high glucose, correlating with enhanced expression of other pro-inflammatory cytokines such as TNFα (106).

**Figure 2:**
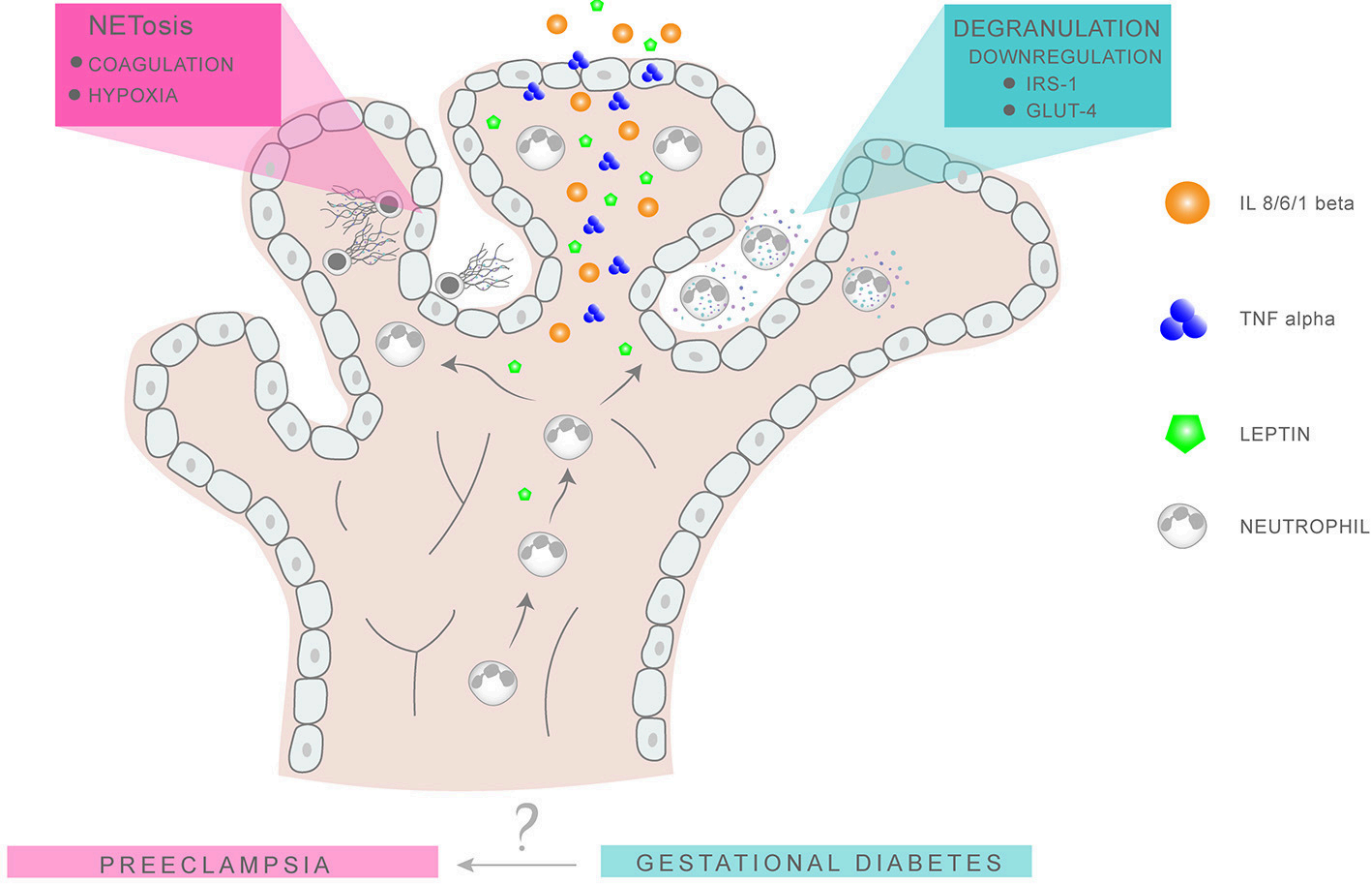
Putative neutrophil activity in GDM and PE placentae. Neutrophil migration into the intervillous space is favored by the chemokine action of leptin, IL-8, and TNFα. In GDM, the release of NE via degranulation results in the degradation of IRS1 and GLUT4 in surrounding tissues, leading to an imbalance in glucose metabolism and insulin response. In PE, excessive NETs formation promotes placental occlusion and hypoxia, leading to extensive tissue damage.

Although there is some evidence that circulating levels of leptin are elevated in advance of detectable glucose intolerance, suggesting that it may be a useful screening marker to detect pregnancies at risk of GDM, these results are currently not conclusive and require further examination [Figure 2; (106, 110)].

In the context of this review it is interesting to note that circulatory leptin levels are also enhanced in PE, being more pronounced in eoPE than loPE cases (111). Akin to GDM, leptin levels increase prior to development of PE symptoms, as suggested by a recent analysis of 387 first trimester samples, of which 120 developed PE (112).

Although no formal proof is present at the moment, these data do suggest that the concomitant expression of leptin and TNFα in GDM placenta, similar to that in PE, could have pronounced effects on neutrophil activity, by promoting migration to this tissue and possibly activation.

## Calprotectin—a new player in diabetes associated coagulopathies

As we have seen above, neutrophils can contribute to diabetes-associated pathologies in a number of ways (81). A recent study has, however, indicated that calprotectin can play a role in the development of diabetes-associated coagulopathies. In their report, Kraakman et al. noted that neutrophils released calprotectin under hyperglycaemic conditions (113). Calprotectin is a dimer of the two calcium binding proteins S100A8 and S100A9 (114). It is abundantly expressed in circulatory neutrophils, where it constitutes up to 60% of the soluble cytosolic protein content. Its main function appears to be an antimicrobial action via the chelation of calcium, zinc, and manganese (114). Calprotectin has been identified as an alarmin, acting as an agonist of TLR4. In this capacity it plays a pivotal role in enhancing inflammatory responses by augmenting other DAMP (damage associated molecular patterns) or PAMP (pathogen associated molecular pattern) signals (114).

In this recent study on experimentally induced murine diabetes, it was determined that calprotectin can bind to the RAGE (receptor for advanced glycation end-products) receptor on liver Kupffer cells, thereby triggering their activation and leading to the production of thrombopoietin (TPO) (113). In the bone marrow, TPO leads to increased platelet production via the activation of megakaryocytes. These freshly released reticulocytes are larger than more mature platelets, contain mRNA and are highly responsive to agonist stimulation. Via the increased expression of p-selectin, activated platelet reticulocytes can readily interact with integrin receptors on leucoytes such as neutrophils, thereby promoting the formation of aggregates (113, 115). These platelet-leucocyte aggregates are a crucial event in the cascade leading to the formation of artherothrombosi (113). In an elegant manner, this study provides a mechanism and possible therapeutic target for diabetes associated cardiovascular disease.

These findings are of considerable interest in the context of PE, a disorder characterized by widespread endothelial dysfunction and cardiovascular damage (18). Consequently, it is worth noting that elevated calprotectin levels were previously noted in pregnancies with GDM and in pregnancies affected by PE Sugulle et al. (116). In addition, elevated expression of calprotectin was noted in placentae from cases with recurrent fetal loss, as well as in hypertensive disorders of pregnancy, most notably in cases with severe PE (117–119).

By a similar mechanism to that observed in T2DM described above (113), it is possible that elevated levels of calprotectin in PE or GDM will stimulate Kupffer cells to produce TPO, thereby leading to an influx of reticulocytes into the maternal circulation. Since neutrophils are primed in normal pregnancy and highly activated in cases with PE (76, 120), these leucocytes will readily interact with activated reticulocytes, thereby providing the basis for pronounced or severe thrombotic lesions so prevalent in this enigmatic disorder.

## Summary and conclusion

Pregnancies complicated by GDM, or other diabetic conditions, are associated with poor outcome and are frequently affected by further complications such as PE, a severe life-threatening condition (6, 31). GDM and especially PE are significant risk factors for adverse post-partum maternal and fetal health. In this review we have attempted to discern whether overt neutrophil activity and NETosis in pregnancies affected by GDM, could contribute to the subsequent development of PE.

Granted the complexity of PE, a condition best described as a syndrome with at least two distinct forms, and the transient nature of GDM, this is an worthy if not impossible task, and it is clear that many factors or facets are missing from a global picture.

In this overview we have highlighted the role of excessive neutrophil activation and NETosis in the etiology of PE, and related these to analogous features evident in GDM. Factors contributing to deregulated neutrophil activity in GDM appear to include hyperglycaemia and the interplay between elevated TNFα levels with a concomitant reduction in its potential regulator A1AT. Of considerable interest are the manifold contributions of NE, especially concerning IRS1, to the pathology of PE and GDM.

Further factors contributing to aberrant neutrophil activation include leptin, whose placental expression is enhanced by high glucose conditions, as well as IL-1β produced via the action of TNFα on adipocytes.

That not all cases with GDM develop PE should serve as a reminder that the described pro-inflammatory features are *per sé* not sufficient to trigger PE, but rather can be viewed as a pivotal components of a cascade, the full dimensions of which are beyond the scope of our current understanding. It is to be hoped that this deficit in our understanding will be changed by the successful implementation of systems biology approaches, thereby finally ushering in the promise of efficacious therapies dreamt of as we entered the post-genomic era. In this manner a significant contribution will be made to ensure optimal long-term fetal and maternal wellbeing.

## Author contributions

SH wrote the manuscript. LV made the figures. LV, SB, XY, EH, NT, PH, OL, IH, SR commented and expanded the manuscript.

### Conflict of interest statement

The authors declare that the research was conducted in the absence of any commercial or financial relationships that could be construed as a potential conflict of interest.
